# Storm‐Petrels Aggregate on the Open Sea to Feast on Opportune Blubber

**DOI:** 10.1002/ece3.72701

**Published:** 2025-12-12

**Authors:** Shahar Dubiner

**Affiliations:** ^1^ School of Zoology, Faculty of Life Sciences Tel Aviv University Tel Aviv‐Yafo Israel

**Keywords:** diet, *Oceanites gracilis*, oil slick, procellariiformes, scavenging, seabirds

## Abstract

Scavenging by seabirds at marine mammal carcasses on the open sea is a poorly documented phenomenon, especially among the smaller species of procellariiformes. During a pelagic survey in the Galápagos, I recorded at least fifty Elliot's storm‐petrels ( *Oceanites gracilis* ) foraging at a large piece of drifting blubber. Individuals pattered at the sea surface to pick up oil droplets, a foraging mode consistent with storm‐petrel ecology yet rarely documented in the context of mammal carcasses. Reports in other species emphasize consumption of blubber or tissue, but this observation suggests that small‐bodied seabirds may instead exploit the more metabolically efficient liquid fraction. This observation raises questions regarding the nutritional role played by opportunistic scavenging in storm‐petrels and the possible sensory cues facilitating such aggregations. Furthermore, it exemplifies the importance of natural history observations in documenting infrequent but ecologically significant interactions.

## Graphical abstract

During a pelagic survey in the Galápagos, I recorded at least fifty Elliot's storm‐petrels ( *Oceanites gracilis* ) foraging at a large piece of drifting blubber. Individuals pattered at the sea surface to pick up oil droplets, a foraging mode consistent with storm‐petrel ecology yet rarely documented in the context of mammal carcasses. This natural history note raises questions regarding the role played by opportunistic scavenging in storm‐petrels and the possible sensory cues facilitating such aggregations.

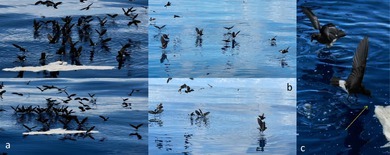

## 1 Nature Note

**FIGURE 1:**
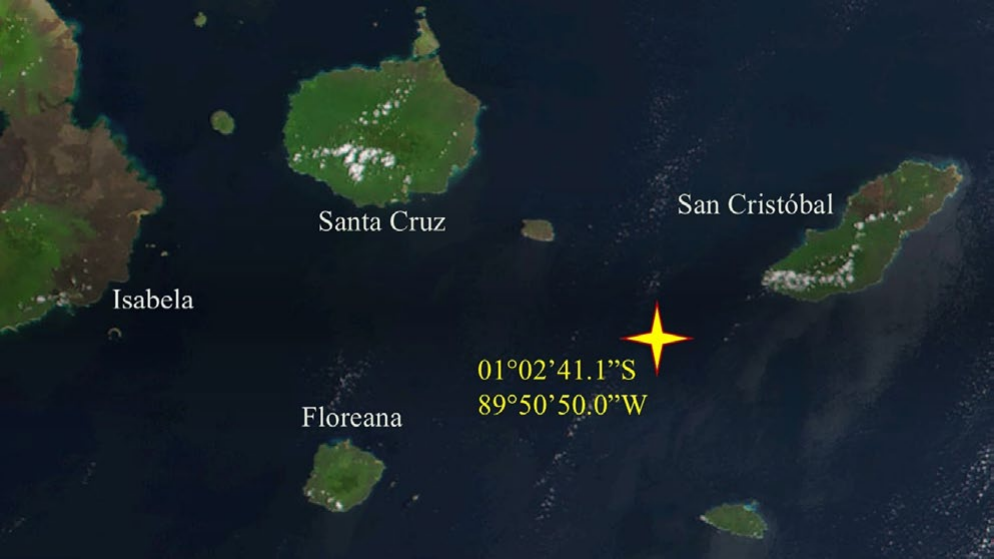
The location of the present observation (01°02′41.1″S, 89°50′50.0″W) shown on a NASA satellite image of southeastern Galápagos (https://visibleearth.nasa.gov/images/57939/galapagos‐islands/57941l). This location has an estimated depth of 340 m and is roughly 120 km from the nearest shore.

**FIGURE 2:**
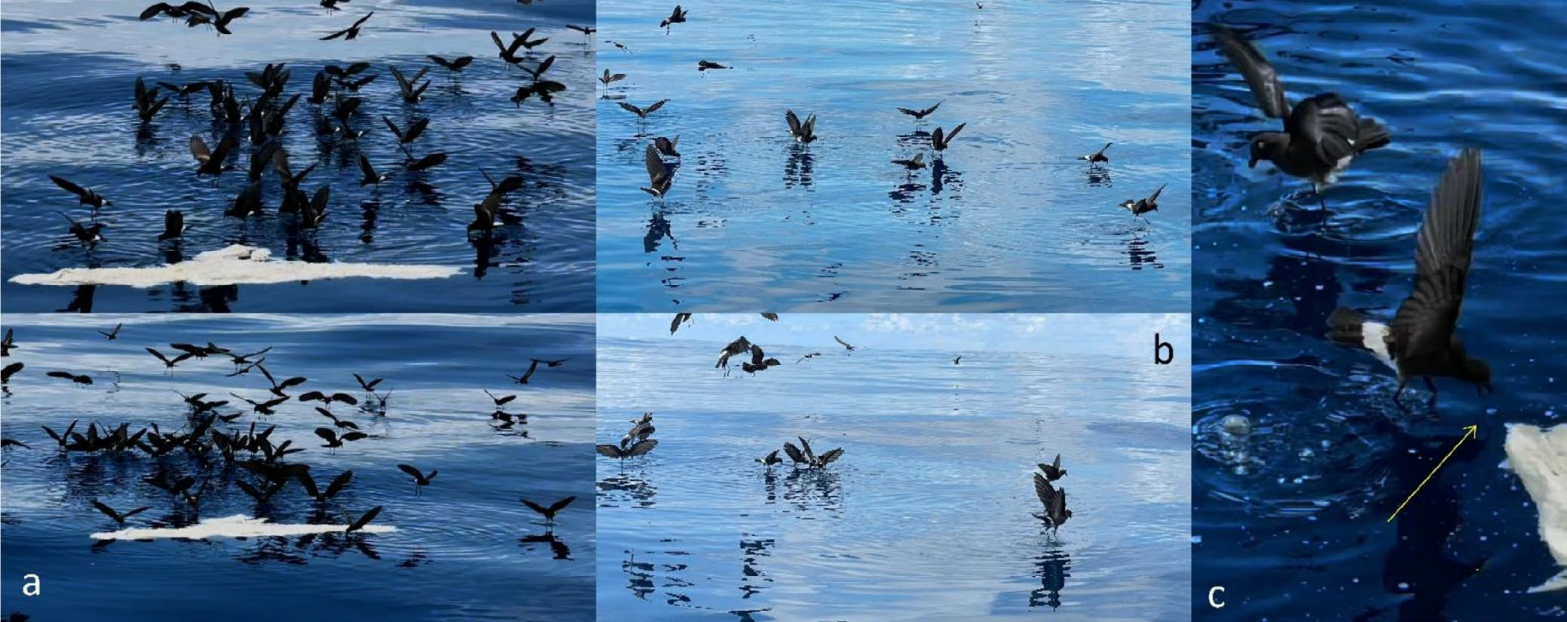
Dozens of *Oceanites gracilis* exhibiting characteristic pattering behavior while foraging on this floating blubber. They did not feed on the solid blubber but around it (a), often several meters away (b). Instead, they peck at the oil slick formed by its decomposition, picking liquid droplets from the sea surface one by one (c, see yellow arrow. Screenshot from 0:17 in Video S2). Photos were taken by Randy Wells and Krystan Wilkinson at 11:05 on the 15th of March 2025.

At first glance, I was perplexed by the scene in front of me. We were out on the open ocean, a hundred and twenty kilometers from the nearest island (Figure 1), yet here was what seemed like a multitude of black butterflies, fluttering at the edge of a gleaming white puddle. Was I becoming delirious from the long hours under the tropical Galápagos sun? Naturally, they turned out to be not butterflies but birds: at least fifty Elliot's storm‐petrels, *Oceanites gracilis* (Procellariformes: Oceanitidae), incessantly pecking at the water surrounding a large piece of drifting blubber (Figure 2a; Video S1). We initially observed the birds from afar with the naked eye, but even as we approached to several meters away, they seemed undisturbed by our presence and continued to feed for many minutes until we were well out of view. The weather was clear that morning (11 AM, 15th of March, 2025), though we had seen heavy rain clouds enveloping the islands in the distance.

Procellariiform seabirds are found in all oceans of the world, especially in the southern hemisphere. The smallest‐bodied species in this group are the storm‐petrels (Cunningham and Nevitt 2005). This group includes *O. gracilis* , a small pelagic bird that is among the most common species around the Galápagos. Its distribution extends as far south as Chile, yet it remains relatively understudied (the “unknown storm‐petrels”; see a recent review on their biology in Delfino and Angarita‐Báez 2024). Despite this general data deficiency, they are commonly understood to feed mainly on planktonic crustaceans and small fishes (as well as mollusks and polychaetes), based on the known diet of similar congeneric species (Croxall et al. 1988; Quillfeldt et al. 2005). Generally, procellariiforms are known for their olfactory sense that can efficiently pick up odors related to krill, fish, and squid (Cunningham and Nevitt 2005), and odors associated with primary productivity over the ocean such as dimethyl sulfide (Nevitt and Veit 1999), which serve as a cue for the presence of planktonic crustaceans and other prey species.

The floating blubber reported herein originated from the carcass of a marine mammal of unknown species. However, judging by its size (a continuous chunk estimated at 150 cm in length), I assume that it belonged to a whale rather than, for example, a sea lion (and definitely not to a planktonic crustacean). Marine mammal carcasses are a known source of occasional food for seabirds, with blubber formerly being amply available due to whaling (Croxall et al. 1988). Scientific notes on this behavior in procellariiform birds mainly come from giant petrels (*Macronectes* spp.) feeding on pinniped and penguin blubber (e.g., González‐Solís et al. 2002, 2003; Gorta et al. 2023; Shaughnessy and Voisin 1979; Van Den Hoff and Newbery 2006). Lévêque et al. (1966) briefly reported witnessing “about 100 storm petrels” feeding at a whale carcass during a 1961 visit to the Galápagos, and Payne et al. (1983) witnessed “several hundred” Wilson's storm‐petrels ( *Oceanites oceanicus* ) feeding on pieces of decayed fatty tissue from the carcass of a dead fin whale in August of 1979. Due to the unlikelihood of routinely observing these rare, opportunistic feeding events on the open sea, their prevalence and importance to storm‐petrels' diet are unknown. In a study on the diet of Leach's storm‐petrel (*Hydrobates leucorhous*), Frith et al. (2020) found marine mammal blubber in only 0.1% of samples, whereas Payne et al. (1983) found whale fat in the proventriculi of two Wilson's storm‐petrels caught immediately after feeding on the carcass. In another study on Wilson's storm‐petrel, Croxall et al. (1988) identified no blubber directly. However, they did find other seal parts (e.g., fur) regurgitated by the birds, and noted that blubber is likely to be digested very quickly and efficiently. Therefore, it may be so unidentifiable when analyzing stomach contents that its contribution to the diet is unknowable.

Contrary to the observations of most aforementioned species, I observed that *O. gracilis* fed while exhibiting pattering behavior (as is typical for all foraging storm‐petrels; Sausner et al. 2016), rather than diving for submerged pieces (Van Den Hoff and Newbery 2006) or briefly alighting on the water to pick food off the surface (Gorta et al. 2023). Furthermore, *O. gracilis* completely ignored the blubber itself, often venturing many meters away (Figure 2b) and targeting individual droplets of oil slick instead (Figure 2c; Video S2). Other seabirds were reported to use the oil slick, a product of decomposition, as merely a cue for locating submerged carcasses (Van Den Hoff and Newbery 2006) but not as a food source. Nevertheless, storm‐petrels were deemed to feed on oil slicks (Gorta et al. 2023; Verheyden and Jouventin 1994). Perhaps due to the small body of storm‐petrels, their beak and digestive system are not adapted to processing large pieces of mammalian tissue. A more plausible explanation, in my opinion, is that the oil slick has a higher content of metabolizable energy, especially since storm‐petrels are known to efficiently digest various lipids (Obst 1986). The birds' small size entails high metabolic rates and a low maximum food intake, and the energy expenditure needed for pattering (while feeding) and long‐distance flights (when arriving at the site of the carcass) is already high. Therefore, metabolizable energy per volume ingested should be maximized (and costs of digestion minimized) during these low‐frequency, unpredictable events. Payne et al. (1983) note that such decaying fat can be quickly converted to stomach oil and carried with a minimum demand for costly water excretion. Oil droplets are energetically superior per volume to the fat‐rich, but highly fibrous and vascularized, blubber tissue.

Since their diet normally relies on planktonic crustaceans, storm‐petrels can smell compounds associated with primary production, and therefore zooplankton abundance, from hundreds of kilometers away (Nevitt and Haberman 2003). The major olfactory cue is dimethyl sulfide (DMS), which triggers them to focus their foraging on the relevant location, where they will be likely to find food, either directly or indirectly—by locating aggregations of feeding seabirds (Nevitt and Veit 1999) or other scavengers (Gorta et al. 2023). Interestingly, storm‐petrels can also detect and locate oil slicks with incredible sensitivity (Verheyden and Jouventin 1994). It will be of great interest to study whether storm‐petrels and other seabirds use olfactory cues beyond the known DMS (which is not produced by mammals) to detect the carcass, or whether they are attracted by DMS produced indirectly by microorganisms during its decomposition.

I do not know which species of marine mammal was the unfortunate donor of this blubber, as we had observed six different species of cetaceans (plus sea lions) during that day and the next. I am also ignorant of how it met its fate, but the waters of the Galápagos archipelago are home to a number of large predators, not least of which is the orca ( *Orcinus orca* ), capable of harming even the largest whales (Totterdell et al. 2022). In a relatively rare appearance (orcas are more common near the western islands), we observed two groups of orcas earlier that day, totaling eleven individuals. It is possible that the storm‐petrels' feast is but the leftovers of another animal's banquet (see also Gorta et al. 2023; on scavenging seabird‐shark interactions). This raises the question: is scavenging restricted to the aftermath of such rare visits by predators, or does it occur more frequently and constitute an important part of their diet? Though leaving many open questions, this natural history note could serve as a glimpse into an intricate foraging association between seabirds, marine mammals, and their predators (Ridoux 1987). The transfer of nutrients and energy—from carcasses to scavengers, from sea to land—is an important ecosystem function. The role of whale carcasses has been studied in the context of deep‐sea ecology (Smith et al. 2015), but the observation reported here highlights their contribution to the food web at, and beyond, the ocean surface as well (see Quaggiotto et al. 2022).

## Author Contributions

**Shahar Dubiner:** conceptualization (lead), investigation (lead), writing – original draft (lead), writing – review and editing (lead).

## Funding

The author was supported by the Azrieli Graduate Studies Fellowship.

## Conflicts of Interest

The author declares no conflicts of interest.

## Supporting information


**Video S1:** Dozens of *Oceanites gracilis* exhibiting characteristic pattering behavior while foraging on this floating blubber. Video was taken by Randy Wells and Krystan Wilkinson at 11:05 on the 15th of March 2025.

**Video S2:** ece372701‐sup‐0002‐VideoS2.MOV. *Oceanites gracilis* did not feed on the solid blubber but around it. Rather, they peck at the oil slick formed by its decomposition, picking liquid droplets from the sea surface one by one (see for example 0:17). Video was taken by Randy Wells and Krystan Wilkinson at 11:05 on the 15th of March 2025.

## Data Availability

This paper contains no data.

## References

[ece372701-bib-0001] Croxall, J. P. , H. J. Hill , R. Lidstone‐Scott , M. J. O'Connell , and P. A. Prince . 1988. “Food and Feeding Ecology of Wilson's Storm Petrel *Oceanites oceanicus* at South Georgia.” Journal of Zoology 216, no. 1: 83–102.

[ece372701-bib-0002] Cunningham, G. B. , and G. A. Nevitt . 2005. “The Sense of Smell in Procellariiforms: An Overview and New Directions.” In Chemical Signals in Vertebrates 10, 403–408. Springer US.

[ece372701-bib-0003] Delfino, H. C. , and J. A. Angarita‐Báez . 2024. “Unraveling the Two Unknown Storm‐Petrels of South America: A Review About the White‐Vented Storm‐Petrel (*Oceanites gracilis*) and Pincoya Storm‐Petrel (*Oceanites pincoyae*).” Marine Biodiversity 54, no. 6: 81.

[ece372701-bib-0004] Frith, R. , D. Krug , R. A. Ronconi , S. N. Wong , M. L. Mallory , and L. A. M. Tranquilla . 2020. “Diet of Leach's Storm‐Petrels (*Hydrobates leucorhous*) Among Three Colonies in Atlantic Canada.” Northeastern Naturalist 27, no. 4: 612–630.

[ece372701-bib-0005] González‐Solís, J. , J. P. Croxall , and D. R. Briggs . 2002. “Activity Patterns of Giant Petrels, *Macronectes spp*., Using Different Foraging Strategies.” Marine Biology 140, no. 1: 197–204.

[ece372701-bib-0006] González‐Solís, J. , J. P. Croxall , and A. G. Wood . 2003. “Sexual Dimorphism and Sexual Segregation in Foraging Strategies of Northern Giant Petrels, *Macronectes halli*, During Incubation.” Oikos 90, no. 2: 390–398.

[ece372701-bib-0007] Gorta, S. B. , B. Brockett , and S. Rapley . 2023. “Interactions Between Seabirds and Sharks at a Fur Seal Carcass.” Marine Ornithology 51, no. 2: 237–241.

[ece372701-bib-0008] Lévêque, R. , R. I. Bowman , and S. L. Billeb . 1966. “Migrants in the Galapagos Area.” Condor 68, no. 1: 81–101.

[ece372701-bib-0009] Nevitt, G. , and R. R. Veit . 1999. “Mechanisms of Prey‐Patch Detection by Foraging Seabirds.” In Proceedings of the 22nd International Ornithology Congress, Durban, edited by N. J. Adams and R. H. Slotow , 2072–2082. BirdLife South Africa.

[ece372701-bib-0010] Nevitt, G. A. , and K. Haberman . 2003. “Behavioral Attraction of Leach's Storm‐Petrels ( *Oceanodroma leucorhoa* ) to Dimethyl Sulfide.” Journal of Experimental Biology 206, no. 9: 1497–1501. 12654888 10.1242/jeb.00287

[ece372701-bib-0011] Obst, B. S. 1986. “Wax Digestion in Wilson's Storm‐Petrel.” Wilson Bulletin 98, no. 2: 189–195.

[ece372701-bib-0012] Payne, P. M. , K. D. Powers , and J. E. Bird . 1983. “Opportunistic Feeding on Whale Fat by Wilson's Storm‐Petrels in the Western North Atlantic.” Wilson Bulletin 95, no. 3: 24.

[ece372701-bib-0013] Quaggiotto, M.‐M. , J. A. Sánchez‐Zapata , D. M. Bailey , et al. 2022. “Past, Present and Future of the Ecosystem Services Provided by Cetacean Carcasses.” Ecosystem Services 54: 101406.

[ece372701-bib-0014] Quillfeldt, P. , R. A. McGill , and R. W. Furness . 2005. “Diet and Foraging Areas of Southern Ocean Seabirds and Their Prey Inferred From Stable Isotopes: Review and Case Study of Wilson's Storm‐Petrel.” Marine Ecology Progress Series 295: 295–304.

[ece372701-bib-0015] Ridoux, V. 1987. “Feeding Association Between Seabirds and Killer Whales, *Orcinus orca* , Around Subantarctic Crozet Islands.” Canadian Journal of Zoology 65, no. 8: 2113–2115.

[ece372701-bib-0016] Sausner, J. , J. C. Torres‐Mura , J. Robertson , and F. Hertel . 2016. “Ecomorphological Differences in Foraging and Pattering Behavior Among Storm‐Petrels in the Eastern Pacific Ocean.” Auk: Ornithological Advances 133, no. 3: 397–414.

[ece372701-bib-0017] Shaughnessy, P. D. , and J. F. Voisin . 1979. “Observations of Giant Petrels *Macronectes* spp. Along the Atlantic Coast.” In Proceedings of the Symposium on Birds of the Sea and Shore, edited by J. Cooper , 199–213. African Seabird Group.

[ece372701-bib-0018] Smith, C. R. , A. G. Glover , T. Treude , N. D. Higgs , and D. J. Amon . 2015. “Whale‐Fall Ecosystems: Recent Insights Into Ecology, Paleoecology, and Evolution.” Annual Review of Marine Science 7, no. 1: 571–596. 10.1146/annurev-marine-010213-135144 25251277

[ece372701-bib-0019] Totterdell, J. A. , R. Wellard , I. M. Reeves , et al. 2022. “The First Three Records of Killer Whales (*Orcinus orca*) Killing and Eating Blue Whales (*Balaenoptera musculus*).” Marine Mammal Science 38, no. 3: 1286–1301.

[ece372701-bib-0020] Van Den Hoff, J. , and K. Newbery . 2006. “Southern Giant Petrels *Macronectes giganteus* Diving on Submerged Carrion.” Marine Ornithology 34: 61–64.

[ece372701-bib-0021] Verheyden, C. , and P. Jouventin . 1994. “Olfactory Behavior of Foraging Procellariiforms.” Auk 111, no. 2: 285–291.

